# Manufacturing of Living Building Materials With Calcifying Cyanobacteria

**DOI:** 10.21769/BioProtoc.5543

**Published:** 2025-12-20

**Authors:** Patrick Jung, Jan Friedek, Laura Briegel-Williams, Miriam Haage-Ott, Carina Neff

**Affiliations:** 1 XCEL – Extreme Cryptogam Ecology Lab, University of Applied Sciences Kaiserslautern, Kaiserslautern, Germany; 2 Microbial Architecture, University of Applied Sciences Kaiserslautern, Kaiserslautern, Germany; 3 Civil Engineering, University of Applied Sciences Kaiserslautern, Kaiserslautern, Germany

**Keywords:** Cement, Concrete, Living building material, Calcification, Cyanobacteria

## Abstract

In recent years, the calcifying properties of some cyanobacteria have been used in the production of living building materials (LBMs), such as bio-concrete, as a CO_2_-friendly alternative for cement. This microbially induced calcium carbonate precipitation (MICP) technique can act as a novel platform technology for carbon capture strategies. Consequently, various research articles have been conducted based on a diverse set of workflows, including several modifications, to manufacture LBMs. However, such articles contain only fragmentary descriptions of the materials and methods used. This protocol is meant to act as a detailed, step-by-step operational manual for the production of LBMs using the cyanobacterial model strain *Picosynechococcus* sp. PCC 7002. The process is divided into several steps, such as the activation of the cyanobacterial-gel solution with CaCl_2_ × 2H_2_O and NaHCO_3_, casting standardized prisms (160 × 40 × 40 mm), and demolding LBMs. Subsequently, bending tensile and compressive strength tests are performed according to the procedures commonly used in concrete and material testing as proof of concept.

Key features

• A comprehensive workflow for the manufacturing of cement-free living building materials with cyanobacteria.

• A cyanobacteria-gelatin-containing solution is activated, mixed with sand, casted, curated, and strength tested.

• Adaptable for other cyanobacterial strains and substitute materials.

## Graphical overview


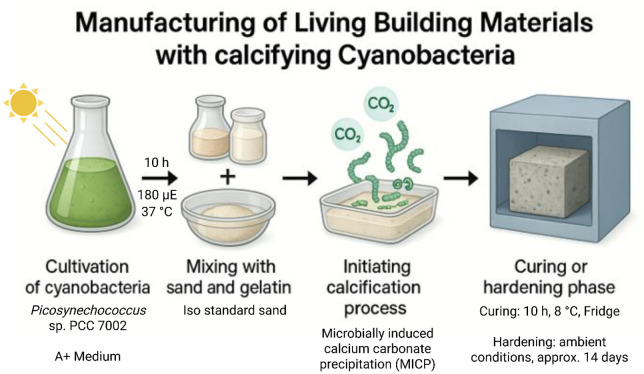


**Simplified scheme for the manufacturing of living building materials (LBMs) such as concrete-like bricks based on the calcification properties of cyanobacteria**

## Background

In view of the ongoing global climate change and associated ecological and economic changes, innovations to reduce emissions are becoming increasingly important. Carbon dioxide (CO_2_) is the primary factor in this context and the key to preventing long-term, irreversible consequences. Over the last 20 years alone, annual CO_2_ emissions have risen by around 45% to an annual 37.12 billion tons [1]. The use of renewable energies and the development of new technologies are the main strategies for achieving the goals of the Paris Climate Agreement and thus a global temperature rise of no more than 1.5–2.0 °C by 2100 [2].

The construction industry plays a decisive role in this. In recent years, the trend shows that the manufacturing sector of the construction industry, in particular, has not been able to achieve any emission reductions [3]. One possible explanation for this could be that adequate substitutes have not yet been developed for the main components in the production of resource-intensive materials. For example, it is not yet possible to produce concrete without cement or entirely from recycled concrete.

Totaling 37% of all CO_2_ emissions, the building sector is one of the main producers of greenhouse gases in the construction industry. Of this, 10% is directly attributable to the construction industry. As a result, around 5%–8% of annual global CO_2_ emissions are attributable to the cement industry, which is therefore seen as a target for optimization. The development of alternative building materials and new recycling methods for more sustainable construction is therefore of great interest for the near future [4].

Research on cyanobacteria is interesting in this context due to their ability to fix CO_2_. During biomineralization, CO_2_ from the environment is bound and supports the precipitation of calcium carbonate (CaCO_3_) under certain circumstances as a consequence of their carbon concentration mechanism (CCM) [5]. This process has already been used to manufacture living building materials (LBMs), and thus, the combination of biotic additives with conventional building materials is currently being researched in order to completely replace cement with microbially induced calcification. Although this has successfully been achieved with cyanobacterial model strains such as *Picosynechococcus* sp. PCC 7002 [6,7], as additives for 3D printing [8] and using other cyanobacterial strains [9], a detailed, general, and comprehensive workflow is missing [10].

In order to provide an extensive methodological description of the workflow for the manufacturing of LBMs with cyanobacteria, we set up this protocol. It is divided into the following steps: i) preparation and incubation of the cyanobacteria-gel solution, ii) casting of the LBMs, iii) de-molding and curation of the LBMs, and iv) bending tensile- and compressive strength tests as a proof of concept. Our protocol describes the general process of manufacturing but also includes alternative steps to broaden the utilization of the protocol for future development of cyanobacterial LBMs.

## Materials and reagents

**Biological materials**

1. *Picosynechococcus* sp. PCC 7002 from Pasteur Culture Collection [11]

**Reagents**

1. NaCl (Merck, CAS: 7647-14-5)

2. MgSO_4_·7H_2_O (Merck, CAS: 7487-88-9)

3. Na_2_EDTA·2H_2_O (Merck, CAS: 6381-92-6)

4. KCl (Merck, CAS: 7447-40-7)

5. CaCl_2_·2H_2_O ((Merck, CAS: 10035-04-8)

6. NaNO_3_ (Sigma-Aldrich, CAS: 7631-99-4)

7. KH_2_PO_4_ (Merck, CAS: 7778-77-0)

8. Trizma base (Merck, CAS: 77-86-1)

9. H_3_BO_3_ (Merck, CAS: 10043-35-3)

10. ZnCl_2_ (VWR Chemicals, CAS: 7646-85-7)

11. MoO_3_ (Merck, CAS: 1313-27-5)

12. Vitamin B12 (Merck, CAS: 68-19-9)

13. FeCl_3_·6H_2_O (Merck, CAS: 10025-77-1)

14. MnCl_2_·4H_2_O (Merck, CAS: 13446-34-9)

15. CuSO_4_·5H_2_O (Merck, CAS: 7758-99-8)

16. CoCl_2_·6H_2_O (Merck, CAS: 7791-13-1)

17. Gelatin (VWR, CAS: 24350.262)

18. ISO standard sand (Normensand.de, CEN-Normsand EN196-1)

19. Oil (any virgin organic rapeseed oil)

20. HCl (Sigma-Aldrich, CAS: 7647-01-0)

21. NaOH (Honeywell Chemicals, CAS: 1310-73-2)

**Solutions**

1. A+ trace components [12] (see Recipes)

2. A+ medium [12] (see Recipes)

3. A+ modified medium [6] (see Recipes)

**Recipes**

**1. A+ trace components [12]**

**Table:** 

Component	Reagent	Stock solution (g/100 mL)	Nutrient solution
1	H_3_BO_3_		3.43 g/L
2	ZnCl_2_		0.0315 g/L
3	MoO_3_		0.003 g/L
4	Vitamin B12		0.0004 g/L
5	FeCl_3_·6H_2_O	3.89	1 mL/L
6	MnCl_2_·4H_2_O	4.3	1 mL/L
7	CuSO_4_·5H_2_O	0.003	1 mL/L
8	CoCl_2_·6H_2_O	0.012	1 mL/L

a. To prepare 1 L of A+ trace components, add 500 mL of dH_2_O to a 1 L glass bottle and add 3.43 g of component 1, 0.0315 g of component 2, 0.003 g of component 3, and 0.0004 g of component 4 as described above while stirring continuously.

b. Prepare 100 mL of each stock solution from components 5–8. Use Milli-Q or dH_2_O for each solution.

c. Then, add 1 mL of each stock solution from components 5–8 above and adjust to 1 L by adding dH_2_O.

d. Store all stock solutions and A+ trace components in a refrigerator.

**2. A+ medium [12]**

**Table:** 

Component	Reagent	Stock solution (g/100 mL)	Nutrient solution
1	NaCl		18 g/L
2	MgSO_4_·7H_2_O		5 g/L
3	Na_2_EDTA·2H_2_O	0.3	10 mL/L
4	KCl	6	10 mL/L
5	CaCl_2_·2H_2_O	3.7	10 mL/L
6	NaNO_3_	10	10 mL/L
7	KH_2_PO_4_	0.5	10 mL/L
8	Trizma base pH 8.2	10	10 mL/L
9	A+ trace components		10 mL/L

a. Prepare 100 mL of each stock solution from components 3–8 from the table above. Use Milli-Q or dH_2_O for each solution.

b. To prepare 1 L of A+ medium, add 500 mL of dH_2_O in a 1 L glass bottle and add 18 g of component 1, 5 g of component 2, and 10 mL of each stock solution of components 3–8 as shown above, while stirring continuously.

*Note: Store all stock solutions in the dark at room temperature.*

c. Fill up to 1 L with dH_2_O and adjust pH to 7.6 with HCl solution.

d. Sterilize by autoclaving (121 °C, 15 psi, 20 min).

e. Add 10 mL of A+ trace components (see Recipe 1) by filter sterilization (0.22 μm).

*Notes:*

*1. A+ trace components is heat sensitive.*

*2. For agar plates, A+ medium is supplemented with sodium thiosulfate pentahydrate (24.819 g/mL) stock solution; 1 mL/L A+ medium and 1.5% (w/v) agarose.*

f. Store the final A+ medium at room temperature before usage.

**3. A+ modified medium [6]**

**Table:** 

Component	Reagent	Stock solution (g/100 mL)	Nutrient solution
1	MgSO_4_·7H_2_O		5 g/L
2	Na_2_EDTA·2H_2_O	0.3	10 mL/L
3	KCl	6	10 mL/L
4	CaCl_2_·2H_2_O	3.7	10 mL/L
5	NaNO_3 _	10	10 mL/L
6	KH_2_PO_4_	0.5	10 mL/L
7	Trizma base pH 8.2	10	10 mL/L
8	A+ trace components		10 mL/L

*Note: NaCl is left out to avoid halite formation.*

**Laboratory supplies**

1. Pipette tips with filter (Mettler Toledo, model: RAININ)

2. Laboratory bottle, 1 L (VWR, catalog number: 215-1517P)

3. Magnetic stirring bar (VWR Collection, catalog number: 442-4521)

4. Sterile indicator strip (Roth, catalog number: XC20.1)

5. Sterile filter 0.22 μm (VWR Collection, catalog number: 514-1266)

6. Plastic syringe 10 mL (Henke Sass Wolf, catalog number: HSWA8300063477)

7. Plastic bottles 50 mL (VWR collection, catalog number: 734-0448)

8. Metal bowls (Roth, catalog number: L942.1)

9. Trowel (Hornbach, catalog number: 8098631)

10. Glass beaker (VWR Collection, catalog number: 213-0480)

11. Nitrile gloves (VWR, catalog number: ANSE93-143/8.5-9)

12. Lids with two olives (VWR Collection, catalog number: SCOT293102807; SCOT1129825)

13. Silicon pipes (Freudenberg Medical Europe GmbH, catalog number: 228-1069/228-1070)

14. Check valves (Brand, catalog number: 229-3311)

15. Spatula (VWR Collection, catalog number: RSGA395.150)

## Equipment

1. Pipettes (Mettler Toledo, model: Pipet-Lite XLS series)

2. Magnetic stirrer/heat function (VWR Collection, catalog number: 442-0661)

3. Autoclave (Fedegari FVG, model: FVG3)

4. Centrifuge (Sigma, catalog number: SIGMA-22017)

5. Clean safety bench (Fedegari FVG, NuAire, model: NU-543)

6. Scientific scales (Sartorius, model: 1712)

7. Precision three-gang molds (form+test prüfsysteme, model: B2709)

8. Fridge (KBS, catalog number: DKU2031)

9. Universal testing machine (ZwickRoell, model: Z100)

10. NanoDrop One (ThermoFisher Scientific, catalog number: ND-ONE-W)

11. Culture cabinet (CLF PlantClimatics/Percival)

12. Full spectral light (MICCYE, model: B0D97RYTGZ)

13. Digital pH measurement device (Mettler Toledo, catalog number: 30046242)

14. Brush (MKK Pinsel, catalog number: 20387)

## Procedure

**A. Cultivation of cyanobacteria**

1. Set the 1 L culture flasks together by cutting silicon pipes into the desired length and mounting them on the olives of the lids. Install the check valves at the intake part and a sterile filter at the outlet olive and close the laboratory glass bottles. Cover the open ends of the silicon pipes with aluminum foil.

2. Sterilize the prepared culture flask by autoclaving (121 °C, 15 psi, 20 min) and let it cool to room temperature.

3. Open the culture flask under sterile conditions and inoculate *Picosynechococcus* sp. PCC 7002 in 500 mL of A+ medium. Close the culture flask after inoculation.

4. Grow *Picosynechococcus* sp. PCC 7002 under discontinuous light illumination (~30 μmol m^-2^·s^-1^; full spectrum; 18/6 h using a time switch) and slight pressurized air supply (using the infrastructure of the institution, e.g., by connecting tubes to an air compressor with oil filter) for mixing at 25 °C until the stationary phase (OD_750_ > 4) is reached.

**B. Preparation of ALS-gel**

1. Collect 400 mL of liquid bacterial culture equilibrated to OD_750_ = 0.3 (sufficient for 2 molds, each containing 3 prisms) under sterile conditions. Centrifuge this at 7,000× *g* for 2 min at room temperature. Discard the supernatant.

2. Fill 400 mL of A+ modified medium in a 1 L laboratory glass bottle and heat it up to 45 °C on a magnetic stirrer under continuous mixing at 300 rpm.

3. Dissolve 40 g of gelatin in the solution mentioned above until a transparent, yellowish, homogenous solution is obtained.

4. Cool the solution down to 40 °C and slowly add 3.36 g of NaHCO_3_.

5. Adjust pH to 7.6 (use HCl to reduce pH and NaOH to raise pH).

6. Add 5.73 g of CaCl_2_·2H_2_O.

**Caution**: Reduce the stirring speed to avoid the formation of foam.

7. Cool the solution down to 37 °C and add the cell pellet.

8. Incubate the solution under continuous, artificial full-spectrum light (180 μmol m^-2^·s^-1^) and moderate stirring for 10 h at 37 °C.

*Note: Set the temperature slightly below 37 °C to avoid overshoot.*

**C. Casting and demolding of the prisms**

1. Apply a thin layer of organic virgin rapeseed oil to the steel three-gang mold using a brush.

2. Add 1.35 kg of ISO standard sand (one package for 1 × 3 prisms) to a metal bowl and gently mix it with 198.50 g of prepared ALS-gel (for 1 × 3 prisms) using a spatula.

**Caution**: To keep a constant grain size distribution regarding the ISO standard sand, prepare the mixture for each 1 × 3 prism mold individually.

*Note: There is a leftover of approximately 50 mL of incubated ALS-gel when preparing 2 × 3 prisms.*

3. Discontinuously mix the ALS-gel-sand by hand at room temperature until it starts to become slightly viscous (e.g., once every 5 min after initial thorough mixing).

**Caution**: Do not mix the ALS-gel-sand too often since air bubbles and the formation of foam negatively influence the stability of the prisms.

4. Fill the mixture into the steel mold by pressing it with the spatula.

5. Leave the mold in the fridge for at least 6 h at 8 °C.

*Note: As the mixture cools down, it physically cross-links and is able to maintain structural integrity, which is sufficient for demolding.*

6. Detach the molds and allow the prisms to dry on metal grates at ambient temperature (20–22 °C; 40%–60% relative humidity; protected from direct sunlight) for at least 14 days and until mass equilibrium has been reached.

**Figure 1.**
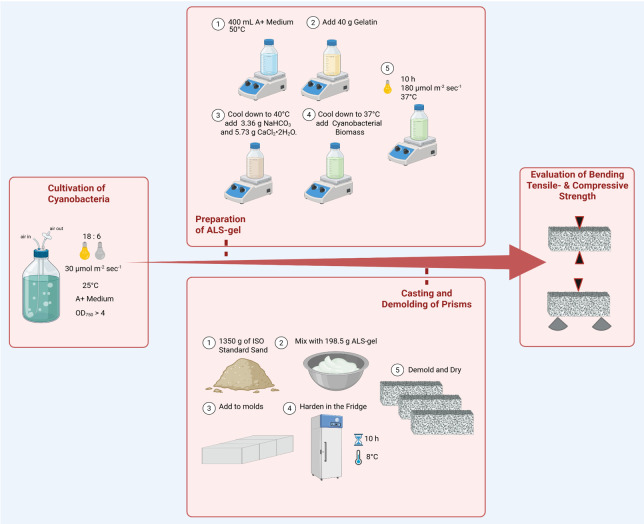
Workflow for manufacturing living building materials (LBMs) with cyanobacteria. Schematic overview including the most relevant steps such as cultivation of cyanobacteria, preparation of ALS-gel, casting and demolding of prisms, and the evaluation of bending tensile and compressive strength.

## Validation of protocol

For the validation of this protocol (Figure 1), six prisms were prepared as described using *Picosynechococcus* sp. PCC 7002, as well as six prisms without cyanobacteria as a control. After no weight loss was detected for the prisms (after 14 days), they were tested for bending tensile and compressive strength. Therefore, each block was mounted on a universal testing machine with an integrated load cell (ZwickRoell) according to DIN EN 196-1:2016-11 (German version of a European standard; this standard sets out the procedure for preparing, curing, and testing mortar specimens made with cement, in order to determine compressive—and optionally tensile—strength). Mechanical properties were tested via three-point bending tensile test (50 ± 10 N/s; load-controlled), followed by uniaxial compressive failure test (2,400 ± 200 N/s) with three replicates each.

**Figure 2.**
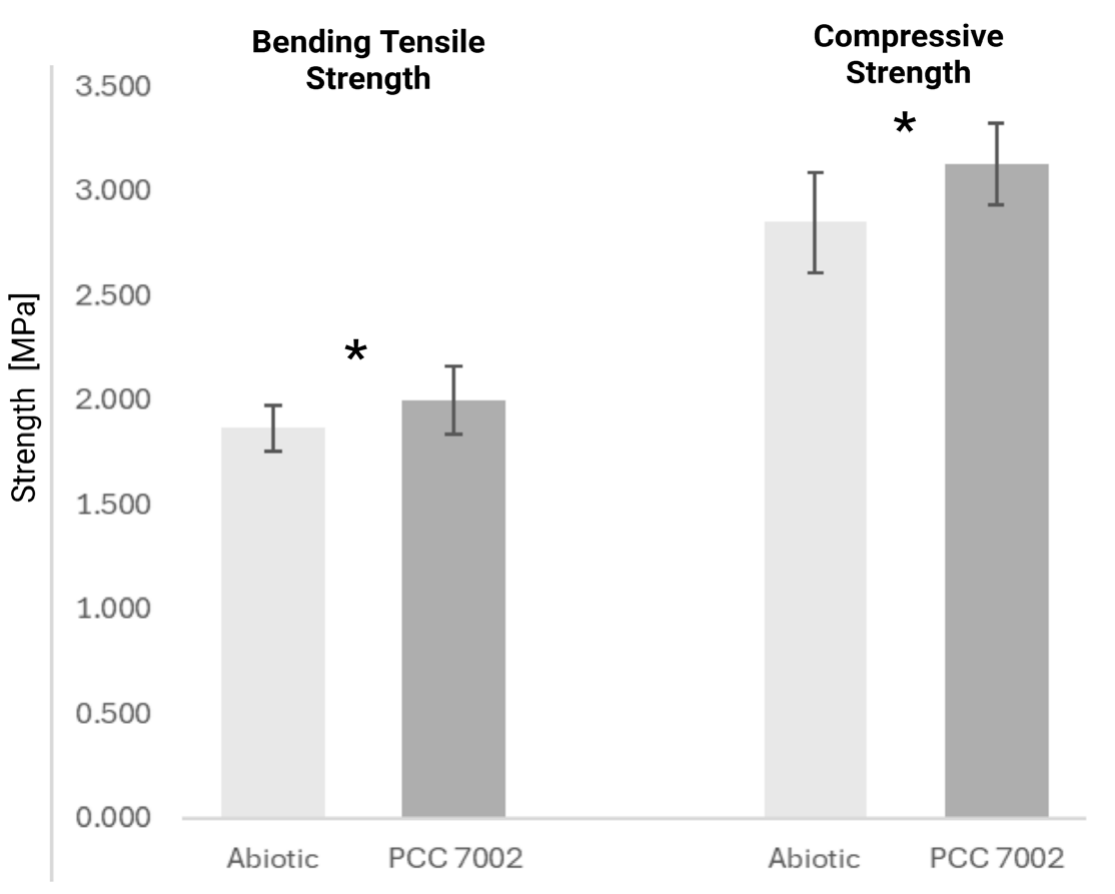
Bending tensile and compressive strengths of three prisms. Independent two-sample *Welch’s* t-test (n = 6, each). * indicates statistical significance.

As a result, a significant difference between the prisms with and without cyanobacteria was detected (Figure 2) for bending tensile strength (t = 2.9717; p = 0.0180) and a highly significant difference for compressive strength (t = 5.75; p = 0.00002). The results show the effect of cyanobacterial calcification on the mechanical properties of the living building materials.

## General notes and troubleshooting

**General notes**

Our protocol is suitable for manufacturing LBMs with compressive strengths, not for regenerative LBMs using old materials from parent LBMs.

**Troubleshooting**

Problem 1: Casted LBMs are not hard.

Possible cause: Gelatin was overheated and/or inoculated longer than described.

Solution: Careful control of the temperature and inoculation time.

Problem 2: LBMs are hollow inside, while the walls are stable.

Possible cause: ALS-gel and sand were mixed too intensively, causing air bubbles, which reduce the stability of the prisms.

Solution: Mix gently by hand only every few minutes.
